# Length of Reproductive Lifespan and Late-Life Cognitive Function Among U.S. Women: Evidence from NHANES 2011-2014

**DOI:** 10.1093/geroni/igaf122.4151

**Published:** 2025-12-31

**Authors:** Julia Unsworth, Hannah Jones, Sahra Sheikhnur

**Affiliations:** OHSU-PSU School of Public Health, Portland, Oregon, United States; OHSU-PSU School of Public Health, Portland, Oregon, United States; OHSU-PSU School of Public Health, Portland, Oregon, United States

## Abstract

Women experience disproportionately higher rates of dementia and cognitive decline than men, and lifetime exposure to sex hormones is hypothesized to contribute to this disparity. Reproductive lifespan, defined as the interval between age at first period (menarche) and menopause, may influence late-life cognitive function through cumulative estrogen exposure, but this relationship remains underresearched in nationally representative U.S. samples. Using cross-sectional data from 1,435 women 60 years and older in the 2011-2014 National Health and Nutrition Examination Survey (NHANES), we examined the relationship between reproductive lifespan and cognitive function. Reproductive lifespan was defined as the duration between age at menarche and age at last menstrual period, categorized into weighted tertiles (≤30, 31-38 years, >38 years). Cognitive function was measured using a composite score derived from the Consortium to Establish a Registry for Alzheimer’s Disease Word Learning and Recall, Animal Fluency Test, and Digit Symbol Substitution Test. Survey-weighted multivariable linear regression models estimated associations, adjusting for age, race/ethnicity, nativity, parity, education, and alcohol use. Women with longer reproductive lifespans (>38 years) reported significantly higher cognitive scores compared to those in the shortest tertile (adjusted β = 0.15; 95% CI: 0.03, 0.26). The association was strongest among college graduates (β = 0.23; 95% CI: 0.03, 0.42), compared to women with lower education with smaller and not statistically significant results. A formal test for interaction confirmed effect modification by education (p = 0.025). These findings suggest that a longer reproductive lifespan is associated with better late-life cognitive function, particularly among women with higher educational attainment.

